# Patient and Visit Characteristics Associated With Use of Direct Scheduling in Primary Care Practices

**DOI:** 10.1001/jamanetworkopen.2020.9637

**Published:** 2020-08-27

**Authors:** Ishani Ganguli, E. John Orav, Claire Lupo, Joshua P. Metlay, Thomas D. Sequist

**Affiliations:** 1 Harvard Medical School, Boston, Massachusetts; 2 Division of General Internal Medicine, Brigham and Women’s Hospital, Boston, Massachusetts; 3 Department of Biostatistics, Harvard T.H. Chan School of Public Health, Boston, Massachusetts; 4 Division of General Internal Medicine, Massachusetts General Hospital, Boston; 5 Mass General Brigham, Boston, Massachusetts

## Abstract

**Importance:**

Medical practices increasingly allow patients to schedule their own visits through online patient portals, yet little is known about who adopts direct scheduling or how this service is used.

**Objective:**

To determine patient and visit characteristics associated with direct scheduling, visit patterns, and potential implications for access and continuity in the primary care setting.

**Design, Setting, and Participants:**

This cross-sectional study used electronic health record (EHR) data from 17 adult primary care practices in a large academic medical center in the Boston, Massachusetts, area. Participants included patients 18 years or older who were attributed in the EHR to an active primary care physician at 1 of the included primary care practices, were enrolled in the patient portal, and had at least 1 visit to 1 of these practices between March 1, 2018, and March 1, 2019, the period of analysis. Data were analyzed from October 25, 2019, to April 14, 2020.

**Main Outcomes and Measures:**

Adoption of direct scheduling, defined as at least 1 use during the study period. Usual scheduling was defined as scheduling with clinic staff by telephone or in person.

**Results:**

We examined 134 225 completed visits by 62 080 patients (mean [SD] age, 51.1 [16.4] years, 37 793 [60.9%] women) attributed to 140 primary care physicians at 17 primary care practices. A total of 5020 patients (8.1% [95% CI, 7.9%-8.3%]) adopted direct scheduling, with an age range of 18 to 95 years. Compared with nonadopters in the same practices, adopters were younger (adjusted odds ratio [AOR] per additional year, 0.98 [95% CI, 0.98-0.99]) and were more likely to be White (AOR, 1.09 [95% CI, 1.01-1.17]) and commercially insured (AOR vs uninsured, 1.40 [95% CI, 1.11-1.76]) and to have more comorbidities (AOR per additional comorbidity, 1.07 [95% CI, 1.04-1.11]). Compared with usually scheduled visits, directly scheduled visits were more likely to be for general medical examinations (1979 visits [36.7%] vs 26 519 visits [21.9%]; *P* < .001) and with one’s own primary care physician (5267 visits [95.2%] vs 94 634 visits [73.5%]; *P* < .001).

**Conclusions and Relevance:**

These findings suggest that direct scheduling was associated with greater primary care continuity. Early adopters were more likely to be young, White, and commercially insured, and to the extent these differences persist as direct scheduling is used more widely, this service may widen socioeconomic disparities in primary care access.

## Key Points

**Question:**

What characteristics are associated with the online portal–based scheduling of medical visits?

**Findings:**

In this cross-sectional study of 62 080 patients and 134 225 completed visits at 17 primary care practices within a large academic medical center, early adopters of direct scheduling were more often young, White, and commercially insured. Compared with visits scheduled by speaking with clinic staff in person or by telephone, directly scheduled visits were more likely to be with one’s own primary care physician.

**Meaning:**

These findings suggest that direct scheduling may contribute to primary care continuity and that if greater adoption by younger, White, commercially insured patients persists, this service may widen socioeconomic disparities in primary care access.

## Teaser

This cross-sectional study examines the patient and visit characteristics associated with online portal-based scheduling of medical visits.

## Introduction

Medical practices increasingly allow patients to schedule their own office visits through online patient portals, otherwise known as *direct scheduling*. This option is offered by the largest electronic health record (EHR) vendors and via external health care applications in the United States and internationally,^1,2,3,4,5,6,7,8^ which is consistent with trends toward self-service in most other industries. In a 2013 survey,^9^ 77% of US respondents said it was important to them that their medical provider offered online appointment booking; however, only 43% of respondents said that direct scheduling was offered in some form at the time.

Direct scheduling is intended to improve patient convenience while reducing administrative burden for practices.^5,10^ This offering may have additional benefits, especially in the primary care setting, such as promoting continuity with one’s usual primary care physician (PCP). Conversely, direct scheduling might worsen disparities in access to care via the so-called digital divide.^8,11,12^ Small studies of individual clinics and hospitals in the US, United Kingdom, Australia, and China provide early evidence that this approach may be associated with lower no-show rates^13,14^ and the ability to schedule visits outside of usual business hours.^15,16^ Yet there is little rigorous research on who uses direct scheduling, how it is used, or any unintended consequences.^17,18^

By January 2018, a large academic medical center in the Boston, Massachusetts, area had introduced direct scheduling across 17 adult primary care practices, providing an opportunity to study early adoption and consequences associated with this approach. We used EHR data to identify patient characteristics associated with direct scheduling adoption, how direct scheduling was used, and potential implications for primary care access and continuity.

## Methods

The Partners HealthCare institutional review board approved this study and waived the use of informed consent per 45 CFR 46.116(f). The study follows the Strengthening the Reporting of Observational Studies in Epidemiology (STROBE) reporting guideline.

### Prestudy Exploratory Survey

To inform our analytical plan, in 2017 and 2018 we purposively sampled 30 clinical and administrative leaders at practices that offered or were planning to offer direct scheduling. We filled in additional details through personal communication with the operational team (eAppendix 1 and eTable 1 in the Supplement).

### Implementation Details

Practices staggered the introduction of direct scheduling between October 2016 and January 2018 (eTable 2 in the Supplement). The direct scheduling service was available through the Epic Systems Corporation patient portal desktop or mobile application and allowed any portal user to schedule a problem-based, follow-up, or annual physical examination appointment in the subsequent 2 to 90 days with any physician or nurse practitioner whom they had previously seen. Users indicated their preferred dates and times, then chose from the time slots made available by the practice or individual clinician. Patients at these practices also had the option of usual scheduling, which involved speaking to a staff member at the front desk by telephone or in person and, in some scenarios, being transferred to a nurse.

Prior to implementation, most practices educated clinicians and staff members about direct scheduling, encouraged patient portal enrollment, and adjusted scheduling templates and front desk workflows to accommodate the new offering. Most practices advertised direct scheduling by sending secure message blasts through the patient portal and displaying flyers or cards in the clinic reception area. Most practices reviewed direct scheduling bookings at least daily to confirm safety and appropriateness.

In the survey, some practice leaders reported concerns that with direct scheduling, patients might schedule unnecessary visits or too many visits at a time. Most practice leaders anticipated benefits, including lower no-show rates, decreased front-desk workload, and improved patient convenience (eTable 1 in the Supplement).

### Data Source

We used the health system’s Enterprise Data Warehouse to extract data from March 1, 2017, to March 1, 2019. The Enterprise Data Warehouse includes patient demographic information collected during registration and detailed clinical encounter and billing data from the EHR.

### Patient and Visit Cohorts

We identified a cohort of adult patients aged 18 years or older who were attributed (defined by the EHR *PCP* field) to an active PCP (a physician with specialty in internal or family medicine providing at least 50 visits during calendar year 2018) at 1 of 17 included primary care practices, were enrolled in the patient portal as of March 1, 2019 (defined by at least 1 portal log-in within the previous 2 years), and had at least 1 completed visit to 1 of these practices between March 1, 2018, and March 1, 2019. We examined all completed visits by these patients to the included practices between March 1, 2018, and March 1, 2019 (the study period), excluding any visits that had been scheduled before a given practice offered direct scheduling (eAppendix 2 in the Supplement).

### Primary Outcome

We defined adopters as patients who scheduled at least 1 primary care visit through direct scheduling during our study period. Nonadopters were defined as those who had at least 1 appointment during the study period but did not use direct scheduling to schedule an appointment.

### Patient Characteristics

For all patients, we captured data on age, sex, race/ethnicity, comorbidity count (range, 0-6; including hypertension, diabetes, chronic kidney disease, depression, asthma, or obesity), and number of within–health system visits (not limited to primary care) and number of within–health system hospitalizations in the year prior to the study period (ie, March 1, 2017, to February 28, 2018). We cross-walked patients’ zip codes to American Community Survey data to measure area-level education (categorized based on percentage of residents with high school education or above) and income (categorized as <200%, 200% to <400%, or ≥400% of the 2017 federal poverty level for a family of 4). Among adopters, we assessed each patient’s number of directly scheduled visits during the study period.

### Practice Characteristics

We determined the number of months each practice had offered direct scheduling prior to the start of the study period (range, 2-17 months). Next, we measured the percentage of patients in each practice who had adopted direct scheduling during the study period.

### Visit Characteristics

We assessed visit characteristics, including whether the visit was usually or directly scheduled, time of day the visit was scheduled, and time elapsed between visit scheduling and occurrence. We ranked the top 30 primary diagnosis codes associated with directly and usually scheduled visits, grouped any codes corresponding to identical diagnoses, reranked the diagnoses, and presented the top 15 diagnoses in each group. We further assessed visit billing level (among those with Evaluation and Management *Current Procedural Terminology* billing codes 99211-99215) and the clinician providing the visit (dichotomized as patient’s attributed PCP vs other).

### Statistical Analysis

We performed descriptive patient-, practice-, and visit-level analyses, including adoption rates overall and by primary care practice. For our primary analysis, we compared direct scheduling adopters vs nonadopters in univariate analyses using the χ^2^ test for categorical data and the *t* test or Wilcoxon test for continuous variables. To identify factors associated with adoption, we built a patient-level multivariable logistic regression model that included patient characteristics and practice fixed effects.

In secondary analyses, we used linear regression to assess the association of the length of time practices had been offering direct scheduling with their patients’ adoption rates. We used the Wilcoxon rank-sum test to compare time from visit scheduling to occurrence and the *t* test and χ^2^ test to compare other characteristics of directly and usually scheduled visits overall and among direct scheduling adopters specifically. We used Poisson regression to analyze the number of visits that adopters had with their own PCPs compared with nonadopters. The model included an offset for each patient’s total number of visits so that it effectively compared the percentage of visits for which patients were seen by their own PCP.

We also created a hierarchical linear probability model to determine the relative magnitude of patient-, physician-, and practice-level components of variance in whether a given visit was usually or directly scheduled. Reported *P* values were 2-sided and considered significant at *P* < .05. All analyses were performed with Stata statistical software version 14.2 (StataCorp). Data were analyzed from October 25, 2019, to April 14, 2020.

## Results

Our study sample included 17 primary care practices, 140 PCPs, 62 080 patients, and 134 225 visits. Patients had a mean (SD) age of 51.1 (16.4) years and included 37 793 (60.9%) women.

### Patient-Level Analysis

**Figure 1.**
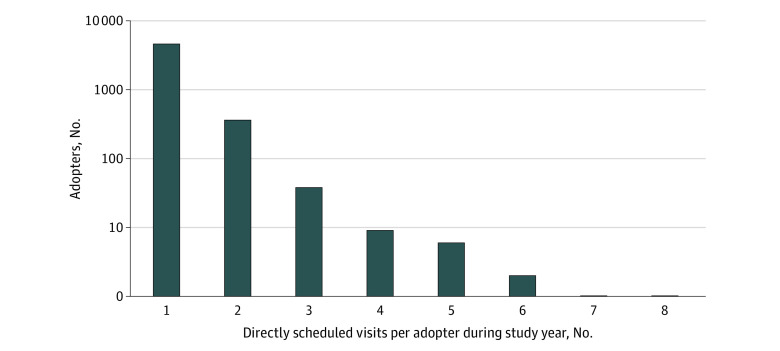
Distribution of Directly Scheduled Visits per Patient Among Direct Scheduling Adopters The y-axis uses a log scale.

Among the 62 080 patients included, 5020 (8.1% [95% CI, 7.9%-8.3%]) used direct scheduling during the study period (age range, 18-95 years) (Table 1). In univariate analyses, adopters, compared with nonadopters, were younger (mean [SE] age, 45.4 [0.2] years vs 51.6 [0.1] years; *P* < .001) and enrolled in commercial insurance (4289 adopters [85.4%] vs 41 264 nonadopters [72.3%]; *P* < .001). Additionally, adopters had fewer comorbidities (mean (SE), 1.03 [0.02] comorbidities vs 1.18 [<0.01] comorbidities; *P* < .001); they resided in areas with lower income (<200% FPL: 217 adopters [4.3%] vs 2018 nonadopters [3.5%]; 200% to <400% FPL: 2836 adopters [56.5%] vs 32 972 nonadopters [57.8%]; ≥400% FPL: 1965 adopters [39.2%] vs 22 014 nonadopters [38.6%]; P < .001) and higher education levels (lowest: 1475 adopters [29.4%] vs 18 691 nonadopters [32.8%]; medium: 1791 adopters [35.7%] vs 19 216 nonadopters [33.7%]; highest: 1754 adopters [34.9%] vs 19 129 nonadopters [33.5%]; *P* < .001). Adopters also had fewer office visits in the prior year (median [interquartile range], 3 [1-7] visits vs 4 [2-8] visits]; *P* < .001) and were less likely to have been hospitalized in the prior year (215 adopters [4.3%] vs 3302 nonadopters [5.8%]; *P* < .001). In the multivariable model with practice fixed effects, using direct scheduling was associated with younger age (adjusted odds ratio [AOR] per additional year, 0.98 [95% CI, 0.98-0.99]), White race (AOR, 1.09 [95% CI, 1.01-1.17]), commercial insurance (AOR vs uninsured, 1.40 [95% CI, 1.11-1.76]), and more comorbidities (AOR per additional comorbidity, 1.08 [95% CI, 1.04-1.11]). Furthermore, having more prior visits was associated with adoption (AOR, 1.00 [95% CI, 1.00-1.01]). Most adopters directly scheduled a total of 1 (4602 patients [91.7%]) or 2 (361 patients [7.2%]) visits during the study period (Figure 1). Adopters had a 11.1% (95% CI 8.8%-13.5%]) higher rate of visits with their own PCPs than nonadopters.

**Table 1.** Characteristics of Direct Scheduling Adopters and Nonadopters

Characteristic	No. (%)		Unadjusted *P* value	Adjusted odds ratio (95% CI)^a^
	Adopters (n = 5020)	Nonadopters (n = 57 060)		
Age, mean (SE), y	45.4 (0.2)	51.6 (0.1)	<.001	0.98 (0.98-0.99)^b^
Sex^c^				
Women	3007 (59.9)	34 786 (61.0)	.14	1 [Reference]
Men	2012 (40.1)	22 273 (39.0)		1.02 (0.96-1.09)
Race				
Other	1054 (21.0)	11 313 (19.8)	.047	1 [Reference]
White	3966 (79.0)	45 747 (80.2)		1.09 (1.01-1.17)^b^
Insurance				
Commercial	4289 (85.4)	41 264 (72.3)	<.001	1.40 (1.11-1.76)^b^
Medicare	402 (8.0)	10 988 (19.3)		0.85 (0.66-1.09)
Medicaid	247 (4.9)	3543 (6.2)		1.17 (0.89-1.52)
Uninsured or missing	82 (1.6)	1265 (2.2)		1 [Reference]
Income, area-level mean of FPL^d^				
<200%	217 (4.3)	2018 (3.5)	.008	1 [Reference]
200% to <400%	2836 (56.5)	32 972 (57.8)		1.02 (0.87-1.19)
≥400%	1965 (39.2)	22 014 (38.6)		1.04 (0.88-1.24)
Area-level proportion of residents with high school education^e^				
Lowest	1475 (29.4)	18 691 (32.8)	<.001	1 [Reference]
Medium	1791 (35.7)	19 216 (33.7)		0.98 (0.90-1.06)
Highest	1754 (34.9)	19 129 (33.5)		1.02 (0.92-1.12)
Comorbidities, mean (SE), No.	1.03 (0.02)	1.18 (<0.01)	<.001	1.08 (1.04-1.11)^b^
Office visits in prior year, median (IQR), No.	3 (1-7)	4 (2-8)	<.001	1.00 (1.00-1.01)^b^
Hospitalized in prior year	215 (4.3)	3302 (5.8)	<.001	0.86 (0.74-1.00)

Abbreviations: FPL, federal poverty level; IQR, interquartile range.

^a^ Multivariable logistic regression model with adoption as the outcome, and including all variables as covariates.

^b^ *P* < .05.

^c^ Data missing for 2 patients.

^d^ Area-level income relative to 2017 FPL for family of 4 (<200% FPL = <$49 200; 200% to <400% FPL = $49 200 to <$98 400; >400% FPL = ≥$98 400). Data missing for 33 patients.

^e^ Lowest, 0% to <90.9%; middle, 90.9% to <95.8%; highest, 95.8% to 100%. Education level missing for 24 patients.

### Practice Analysis

Adoption rates differed widely across practices (range, 1.3%-18.9%). Practices that had been offering direct scheduling for a longer time had higher practice-wide adoption rates during the study period (0.93% [95% CI, 0.44%-1.41%] increase in practice adoption for every additional month of direct scheduling being offered) (eFigure 1 in the Supplement).

### Visit Analysis

**Figure 2.**
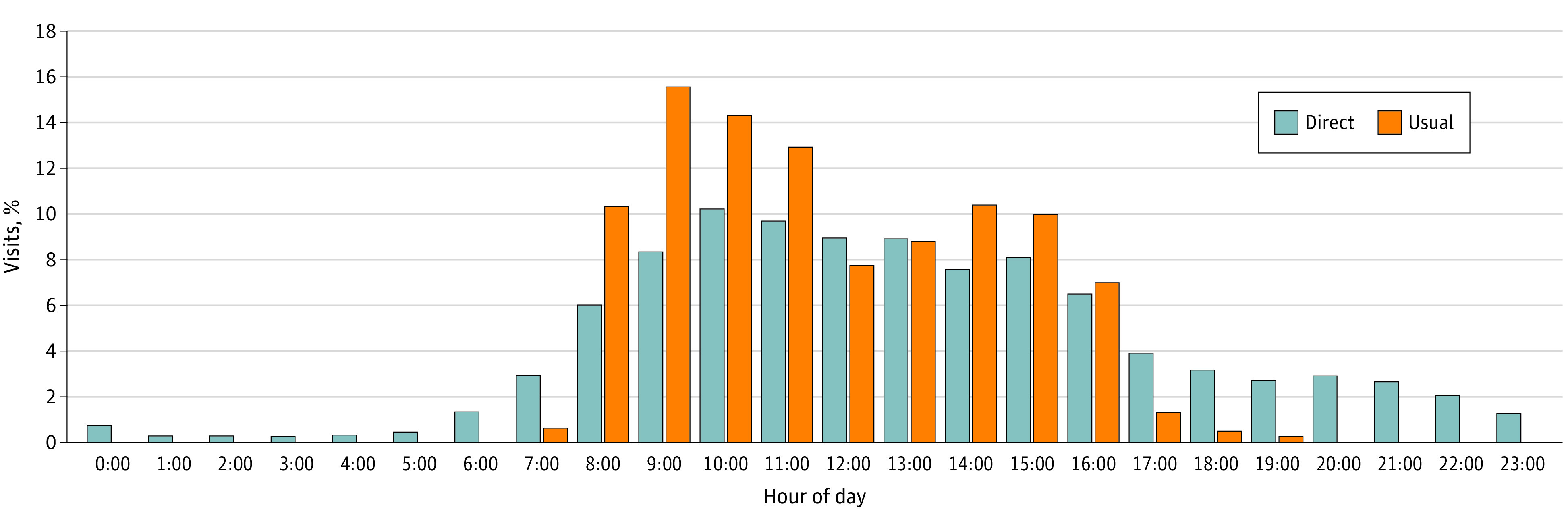
Time of Day Visit Scheduled for Directly vs Usually Scheduled Visits

During the study period, 5531 of 134 225 visits (4.1% [95% CI, 4.0%-4.2%]) were directly scheduled. Of directly scheduled visits, 4331 visits (78.3%) were scheduled during usual business hours (ie, 8 am-5 pm) (Figure 2). Directly scheduled visits had longer wait times from scheduling to occurrence (median [interquartile range], 20 [7-51] days vs 14 [1-66] days; *P* < .001). At the visit level, directly scheduled visits were more likely than usually scheduled visits to carry a primary diagnosis of general medical examination (1979 visits [36.7%] vs 26 519 visits [21.9%], *P* < .001) (Table 2). Among problem-based visits for established patients, directly scheduled visits were less likely than usually scheduled visits to be billed at the highest complexity level (Evaluation and Management code 99215) (80 visits [3.1%] vs 4083 visits [5.1%]; *P* < .001) (eFigure 2 in the Supplement). Directly scheduled visits were more likely than usually scheduled visits to be with the patient’s own PCP (5267 visits [95.2%] vs 94 634 visits [73.5%]; *P* < .001).

**Table 2.** Distribution of Most Common Primary Diagnoses Among Directly Scheduled and Usually Scheduled Visits

Rank	Primary diagnosis	No. (%)^a^
**Directly scheduled visits (n = 5390)**		
1	General medical examination	1979 (36.7)
2	Hypertension	252 (4.7)
3	Hyperlipidemia	109 (2.0)
4	Diabetes	59 (1.1)
5	Anxiety	58 (1.1)
6	Immunization	55 (1.0)
7	Hypothyroidism	50 (0.9)
8	Gastroesophageal reflux disease	46 (0.9)
9	Cough	41 (0.8)
10	Palpitations	36 (0.7)
11	Low back pain	35 (0.6)
12	Malaise	30 (0.6)
13	Cervicalgia	29 (0.5)
14	Immune issue	27 (0.5)
15	Vitamin D deficiency	27 (0.5)
**Usually scheduled visits (n = 120 886)**		
1	General medical examination	26 519 (21.9)
2	Hypertension	8637 (7.1)
3	Upper respiratory infection	2947 (2.4)
4	Diabetes	2840 (2.3)
5	Immunization	2271 (1.9)
6	Cough	2178 (1.8)
7	Hyperlipidemia	1447 (1.2)
8	Knee pain	1120 (0.9)
9	Low back pain	1097 (0.9)
10	Hypothyroidism	1003 (0.8)
11	Anxiety	927 (0.8)
12	Rash	893 (0.7)
13	Dizziness	757 (0.6)
14	Gastroesophageal reflux disease	693 (0.6)
15	Opioid dependence	688 (0.6)

^a^ There were a total of 4720 unique primary diagnosis codes represented in the visit sample. Percentages are calculated among 126 276 of 134 225 visits that had a diagnosis code available.

Among adopters specifically, 5531 of 11 434 visits (48.4% [95% CI, 47.5%-49.3%]) were directly scheduled. When we compared directly scheduled to usually scheduled visits among adopters, directly scheduled visits were still more likely to carry a primary diagnosis of general medical examination (1979 visits [36.7%] vs 792 visits [14.2%]; *P* < .001) and more likely to be with the patient’s own PCP (4103 visits [95.2%] vs 5267 visits [69.5%]; *P* < .001). In the hierarchical model, patient factors accounted for 22.0% of the variance in use of direct vs usual scheduling, more than practice (2.5%) or PCP (1.0%) factors, while 74.6% of the variance remained unexplained.

## Discussion

This cross-sectional study across 17 large primary care practices offering direct scheduling found that early adopters of the patient portal feature were more likely to be younger and White and to have commercial insurance, more comorbidities, and higher prior utilization compared with nonadopters in the same practices. Prior work has reported mixed results on sex and age.^13,14,19,20^ Most directly scheduled visits were scheduled with a patient’s own PCP and during usual business hours. Compared with nonadopters, direct scheduling adopters had higher rates of visits with their own PCP.

Our results suggest that direct scheduling may contribute to continuity and access, core aspects of high-functioning primary care that are associated with better health outcomes and lower costs.^21,22,23^ Directly scheduled visits were more likely than usually scheduled visits to take place with a patient’s own PCP, even when comparing these visit types among direct scheduling adopters, likely in part due to the design feature requiring patients to schedule their appointment with a clinician they had seen before. We also found evidence that patients might find direct scheduling more convenient than usual scheduling: most directly scheduled visits were scheduled during usual business hours when patients could have called the office yet chose to schedule online. This finding substantiates concerns that usual scheduling processes can be prohibitively cumbersome—by one estimate, it took a mean of 8.1 minutes to schedule a doctor’s appointment by telephone, with calls transferred 63% of the time.^24^

At the same time, our findings raise the possibility that direct scheduling might contribute to disparities in primary care access. As direct scheduling is more widely adopted, its disproportionate use among younger, White, commercially insured patients may crowd out visit access for older patients and patients in racial/ethnic minority groups with potentially greater need for, or historically decreased access to, primary care.^25,26^ These findings are particularly worrisome given that we restricted our sample to patients who had enrolled in the patient portal. Prior studies show that members of racial/ethnic minority groups and other historically underserved populations are less likely to enroll in patient portals in the first place.^8,11^ Even among patients with portal access, those who are socioeconomically disadvantaged are less likely to use the technology.^8,27^

Furthermore, while practice leaders’ concerns about patients directly scheduling too many visits were largely unfounded (99% of adopters scheduled just 1 or 2 visits in a year), our results leave open the possibility of more unnecessary visits. Directly scheduled visits were less likely than usually scheduled visits to be billed at the highest complexity level. They were also more likely to represent general medical examinations (partly a consequence of the design), which are of uncertain value among younger, healthy adults.^28^ More reassuringly, direct scheduling adopters had slightly more comorbidities and therefore, perhaps greater need for the service, consistent with prior work showing higher rates of comorbidities among portal adopters in general.^11^

In addition to continuity and access considerations, direct scheduling theoretically offers a third benefit of practice efficiency. While we did not directly examine office staff workload or use of clinician schedules in this study, more recent (ie, July-September 2019) internal operational data of all visits from these 17 practices show that directly scheduled visits had higher cancellation rates than usually scheduled visits (4-57 percentage points higher than for usually scheduled visits across practices), consistent with the possibility that patients may work the schedule by canceling and rescheduling as new options arise or their personal schedules change. For all but 1 of 17 practices, these operational data show directly scheduled visits had the same or lower no-show rates (0-8 percentage points fewer no-show visits) (Mariela Arnal Istillarte, MSIE [Massachusetts General Hospital] email, December 2, 2019).

### Limitations

This study has several limitations. It examines a single academic medical center, albeit a large center with 17 primary care sites covering more than 65 000 patients. We required study participants to be enrolled in the patient portal, since this is a prerequisite to using the direct scheduling application. Of note, internal, operational data show that 50% of primary care patients across the larger health system were active portal users as of December 2019 and that these users were more often women, English-speaking, and White compared with nonusers (Sarah Wilkie, MS [Mass General Brigham], email, January 6, 2020; eTable 3 in the Supplement). There was heterogeneity among practices, but we accounted for this using practice fixed effects. Additionally, any interpretation of statistical significance should be cautious of false-positive results due to multiple testing.^29^

## Conclusions

The findings of this cross-sectional study suggest that that direct scheduling may promote continuity and convenience but also has the potential to widen disparities in primary care access. Since widespread adoption of direct scheduling is likely inevitable, in keeping with other service industries, it will be important to evaluate any associations with health outcomes over time.

## Notes

Supplement. **eAppendix 1.** Prestudy Exploratory Survey **eTable 1.** Survey of Primary Care Practice Leaders on Anticipated and Observed Outcomes of Direct Scheduling **eTable 2.** Implementation Schedule **eAppendix 2.** Data Validation **eFigure 1.** Direct Scheduling Adoption Rates Within Practices, by Time Since Adoption **eFigure 2.** Distribution of Evaluation and Management Billing Codes Among Usually and Directly Scheduled Visits **eTable 3.** Characteristics of Patient Portal Users Among Partners Healthcare Primary Care Patients in 2019
