# Supplementary material for: Patient and Visit Characteristics Associated With Use of Direct Scheduling in Primary Care Practices
Source: JAMA Netw Open. 2020 Aug 27;3(8):e209637. doi: 10.1001/jamanetworkopen.2020.9637 (PMC7453311; doi:10.1001/jamanetworkopen.2020.9637)
Supplement: Supplement. — eAppendix 1. Prestudy Exploratory Survey eTable 1. Survey of Primary Care Practice Leaders on Anticipated and Observed Outcomes of Direct Scheduling eTable 2. Implementation Schedule eAppendix 2. Data Validation eFigure 1. Direct Scheduling Adoption Rates Within Practices, by Time Since Adoption eFigure 2. Distribution of Evaluation and Management Billing Codes Among Usually and Directly Scheduled Visits eTable 3. Characteristics of Patient Portal Users Among Partners Healthcare Primary Care Patients in 2019 [file jamanetwopen-3-e209637-s001.pdf]

Supplementary Online Content

Ganguli I, Orav EJ, Lupo C, Metlay JP, Sequist TD. Patient and visit characteristics associated with use of direct scheduling in primary care practices. *JAMA Netw Open*. 2020;3(8):e209637. doi:10.1001/jamanetworkopen.2020.9637

eAppendix 1. Prestudy Exploratory Survey

eTable 1. Survey of Primary Care Practice Leaders on Anticipated and Observed Outcomes of Direct Scheduling

eTable 2. Implementation Schedule

eAppendix 2. Data Validation

eFigure 1. Direct Scheduling Adoption Rates Within Practices, by Time Since Adoption

eFigure 2. Distribution of Evaluation and Management Billing Codes Among Usually and Directly Scheduled Visits

eTable 3. Characteristics of Patient Portal Users Among Partners Healthcare Primary Care Patients in 2019

This supplementary material has been provided by the authors to give readers additional information about their work.

© 2020 Ganguli I et al. *JAMA Network Open.*

eAppendix 1. Prestudy Exploratory Survey

To inform our analytic plan, we purposively sampled 30 clinical and administrative leaders at primary care practices that offered or were planning to offer direct scheduling in 2017-2018. We administered a mixed mode (online and paper) survey instrument to capture how practices used or planned to use direct scheduling. This 15-item survey included questions on existing processes to book appointments, pre-implementation preparation, patient outreach approaches, implementation strategies, and anticipated and observed effects of the program.

© 2020 Ganguli I et al. *JAMA Network Open.*

eTable 1. Survey of Primary Care Practice Leaders on Anticipated and Observed Outcomes of Direct Scheduling

Potential outcomes	Anticipated, N=30 (%)	Observed, N=30 (%)
Patients schedule unnecessary visits	12 (40)	0 (0)
Patients schedule too many visits at a time	6 (20)	0 (0)
Lower rates of no-shows and last-minute cancellations	17 (57)	2 (7)
Decreased front desk workload	20 (67)	1 (3)
Improved patient convenience	17 (57)	1 (3)

© 2020 Ganguli I et al. *JAMA Network Open.*

eTable 2. Implementation Schedule

Go-live year, month		Practice
2016	October	MGH Back Bay HealthCare Center
2017	May	Mass General West Medical Group, Ambulatory Practice of the Future, MGH Downtown
	June	MGH Beacon Hill Primary Care, MGH Everett Family Care
	September	Massachusetts General Medical Group, Bulfinch Medical Group
	October	Primary Care Associates, Revere HealthCare Center (2), Broadway Primary Care - Revere
	December	Women's Health Associates
2018	January	Chelsea HealthCare Center (2), Charlestown HealthCare Center, Primary Care Assembly Row

© 2020 Ganguli I et al. *JAMA Network Open.*

eAppendix 2. Data Validation

We used the EPIC "PCP" assignment field to determine a patient's primary care physician. This field is used for a host of operational purposes (e.g., to notify the PCP of an emergency department visit, to forward specialist consultation notes, and to attribute quality improvement metrics and bonuses). It is updated regularly to reflect the patient's, physician's, and primary care office's understanding of the patient's true PCP.

Patient medical record numbers (MRNs) were matched to EPIC Patient IDs based on all available patient identifiers. We eliminated 3 MRNs and 5 visits for which we could not reliably match MRN to unique patient (match probability <100%). We eliminated 6727 visits that were scheduled before a given practice had adopted direct scheduling and 3,473 patients who no longer met our inclusion criteria as a result of these eliminations.

© 2020 Ganguli I et al. *JAMA Network Open.*

eFigure 1. Direct Scheduling Adoption Rates Within Practices, by Time Since Adoption

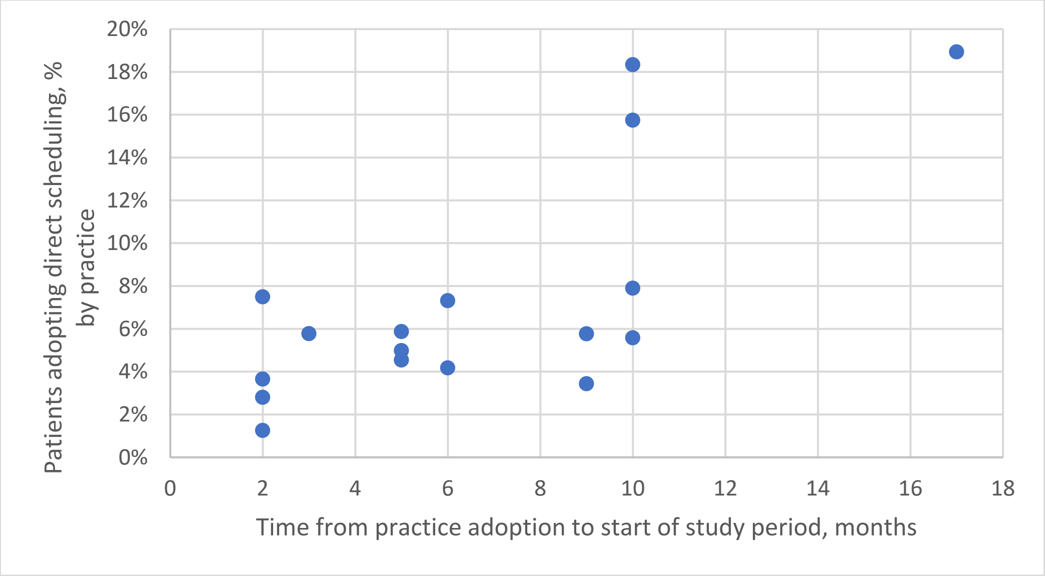

Each dot in this figure represents one of the 17 primary care practices in the study.

© 2020 Ganguli I et al. *JAMA Network Open.*

eFigure 2. Distribution of Evaluation and Management Billing Codes Among Usually and Directly Scheduled Visits

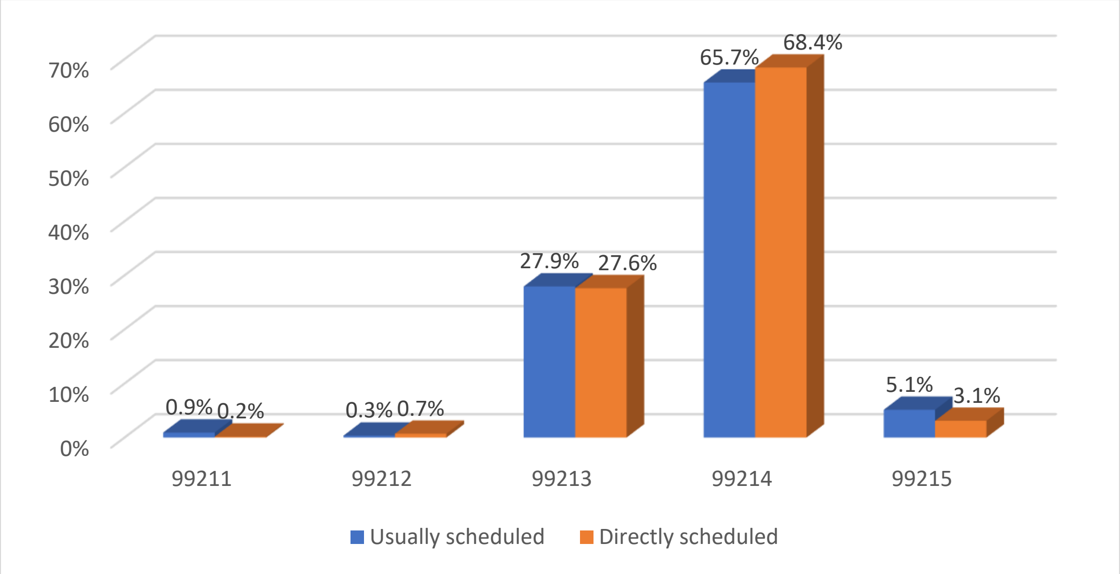

E&M = evaluation and management. Higher billing codes represent longer or more complex visits. For this analysis, we examined the 79,674 (62%) of 128,694 usually scheduled visits and 2,585 (47%) of 5531 directly scheduled visits that were billed as 99211-5.

© 2020 Ganguli I et al. *JAMA Network Open.*

eTable 3. Characteristics of Patient Portal Users Among Partners Healthcare Primary Care Patients in 2019

Characteristic		Portal users N=465,005 (%)	Non-portal users N=459,010 (%)
Female		278,037 (59.8)	239,117 (52.1)
Primary language	English	447,719 (96.3)	390,372 (85.0)
	Spanish	4,013 (0.9)	34,626 (7.5)
	Other	13,273 (2.9)	34,012 (7.4)
Race	White	380,614 (81.9)	324,507 (70.7)
	Black	16,972 (3.6)	30,368 (6.6)
	Asian	26,891 (5.8)	18,104 (3.9)
	Hispanic	4,002 (0.9)	11,194 (2.4)
	Other	36,526 (7.9)	74,837 (16.3)

© 2020 Ganguli I et al. *JAMA Network Open.*
